# Hidden for years: Torsion and infarction in the wandering spleen of a 45-year-old woman

**DOI:** 10.1016/j.ijscr.2024.110092

**Published:** 2024-08-03

**Authors:** Alsadig Suliman, Mohamed Soud, Hussein Elfaki, Amel Mohamed, Muntasir Mukhatar, Hiba Suliman

**Affiliations:** a Department of General Surgery, Sudan Medical Specialization Board, Khartoum, Khartoum, Sudan; b University of Gezira, Wad Madni, Sudan; c Department of General Surgery, Khartoum police Hospital, Khartoum, Khartoum 21111, Sudan; d Wad Medani Teaching Hospital, Department General Surgery, Wad Medani, Sudan; e Wad Medani College Of Medical Sciences & Technology

**Keywords:** Wandering spleen, Case report, Splenectomy, Torsion

## Abstract

**Introduction and importance:**

Wandering spleen (WS), characterized by abnormal mobility due to laxity of its ligaments, is a rare condition often presenting diagnostic challenges. Its complications, such as torsion and infarction, necessitate prompt recognition and management to prevent life-threatening outcomes.

**Case presentation:**

We report a case of a 45-year-old female presenting with acute abdominal pain, constipation, and a palpable mass in the right iliac fossa. Imaging revealed a torsion of a wandering spleen, a rare occurrence exacerbated by delayed diagnosis despite a decade of symptoms.

**Clinical discussion:**

Diagnostic imaging, including abdominal ultrasound and CT scan, played a crucial role in confirming the ectopic spleen and guiding surgical intervention. Immediate laparotomy revealed a twisted spleen necessitating splenectomy due to non-viability post-detorsion attempt.

**Conclusion:**

This case underscores the importance of considering wandering spleen in the differential diagnosis of acute abdominal pain, especially in patients with chronic intermittent symptoms. Surgical intervention remains the definitive treatment, emphasizing the risks associated with conservative management in preventing complications.

## Highlights

• Rare case of torsion of a wandering spleen in a 45-year-old female, underscores the challenges of diagnosing a rare condition with non-specific symptoms over a prolonged period.
• Successful open splenectomy performed following 1440° clockwise torsion and unsuccessful detorsion attempt.
• Emphasis on the diagnostic role of imaging (CT scan) in identifying ectopic spleen and guiding surgical intervention.
• Importance of clinical suspicion for wandering spleen in patients with chronic abdominal symptoms.
• Surgical management as the gold standard due to high risk of complications with conservative approaches.

## 1 Introduction

Wandering spleen (WS) also known as ectopic spleen is a rarely diagnosed clinical entity, with an incidence of 0.2 % [1]. Fewer than 500 cases have been reported in the literature [2]. This uncommon clinical condition is characterized by increased laxity or total absence of the splenic ligaments which prevents the spleen from maintaining its normal position, causing increased mobility [3]. WS is congenital or acquired with no genetic background and is caused by the absence, maldevelopment, or laxity of one or more splenic ligaments that keep the spleen static in the left hypochondrium [4]. Because of this abnormal anatomical condition, the vascular pedicle is usually elongated and mobile, allowing its torsion and leading to splenic infarction [5].

## 2 Methods

This work has been reported in line with the SCARE criteria [6].

## 3 Case presentation

A 45-year-old woman presented to the emergency room complaining of lower abdominal pain for two weeks that had worsened over the previous three days. Additionally, she reported nausea, intermittent fever, vomiting, and constipation. Her pain was temporarily relieved with analgesia but aggravated by physical activity. She denied any recent trauma, and her drug and family history were unremarkable.

Her vital signs were as follows: pulse rate of 102 beats/min, blood pressure of 100/70 mmHg, respiratory rate of 21/min, temperature of 37.8 °C, and SpO2 of 97 %. Upon physical examination, her abdomen was mildly distended and contained a mobile, smooth, 20 × 10 cm mass in her right lower abdomen that extended into her pelvis. This mass was tender to the touch, movable in all directions, and able to go both above and below it. Laboratory findings showed a TLC count of 11,000/mm^3^ (normal range 4000–11,000), a neutrophil count of 78 % (normal range 40–75 %), and a hemoglobin count of 11.3 g/dl (normal range 11.5–16.5 g/dl). Her INR was 1.2, and her blood type was B+. She tested negative for anti-HCV Ab, HBsAg, and HIV.

**Fig. 1:**
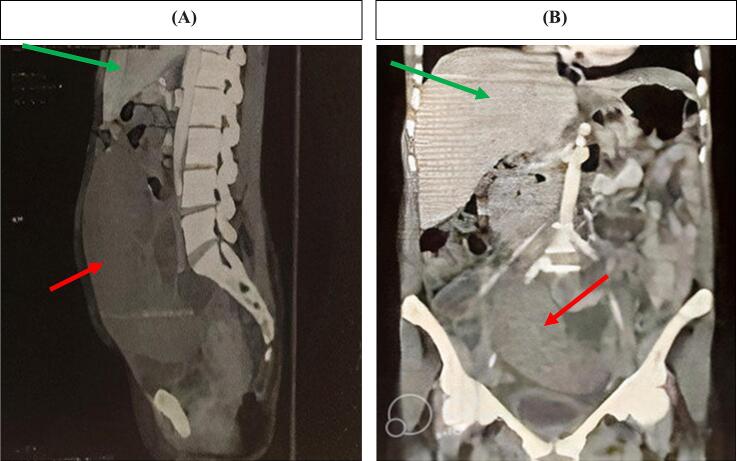
The sagittal (A) and frontal axis (B) in this woman showed the spleen in pelvic activity (red arrow) Liver in (green arrow). (For interpretation of the references to colour in this figure legend, the reader is referred to the web version of this article.)

**Fig. 2:**
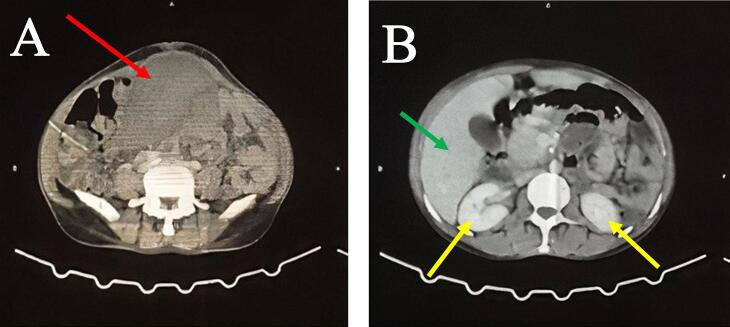
The axial CT image from (A) and (B) reveals that the spleen (red arrow) is not located in its usual position. It is situated at a lower level of the kidneys (yellow arrow) and in the pelvic cavity. Liver in (green arrow). (For interpretation of the references to colour in this figure legend, the reader is referred to the web version of this article.)

An abdominal ultrasound revealed that her spleen was not located in its usual position (left hypochondrium) but was instead present in the central abdominal region, extending to the suprapubic area, with a size of 25 cm and a central echogenic area and fluid collection surrounding it. An urgent abdominal CT scan, both with and without contrast, showed an enlarged ectopic spleen measuring 25.5 cm in length and 13 cm in width, located in the mid and lower abdomen and extending into the pelvis (Fig. 1). The splenic fossa was empty, and no splenic enhancement was observed after intravenous contrast administration (Fig. 2).

**Fig. 3:**
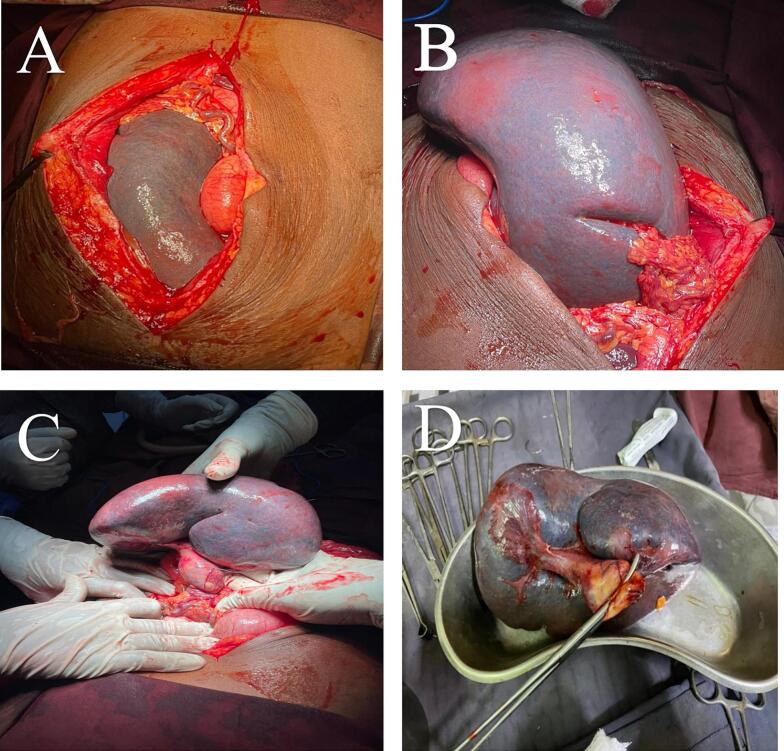
A B C An Intraoperative image displaying splenomegaly and splenic pedicle torsion in the context of wandering spleen pathology. D: Resected specimen.

Based on the patient's vital signs, clinical examination, and imaging studies, it was necessary to immediately take the patient to the operating room. Due to equipment limitations and a high suspicion of peritonitis, laparotomy was necessary instead of laparoscopic surgery. Upon opening the abdomen, a significantly enlarged, and congested spleen was observed in the central abdomen with an abnormally elongated pedicle and twisted 1440° clockwise. However, after counterclockwise detorsion, despite administering 100 % oxygen and wrapping the spleen with heated gauze, it remained non-viable. As a result, a decision was made to perform a splenectomy. The operation passed without complication, and the patient recovered smoothly from anesthesia and was sent to the ward in stable condition (Fig. 3). The splenectomy procedure was performed by a surgical team led by a consultant, who is a board-certified general surgeon with extensive experience in abdominal surgeries, including splenectomies.

The postoperative course was uneventful, and the patient was discharged on the 4th postoperative day without any complications. Two weeks after surgery, she was vaccinated against Pneumococcus, Meningococcus, and *Haemophilus influenzae*.The macroscopic examination showed the resected spleen weighed approximately 1154 g and measured 25.5 cm in length and 13.5 cm in width. While Histopathological assesment showed an expansion of the red pulp, extensive ischemic necrosis, fibrosis, and subcapsular hemorrhage. No evidence of neoplasia was detected.

## 4 Discussion

The spleen is the largest organ in the reticuloendothelial system, weighing normally between 150 and 200 g and measuring 10.9 ± 1.4 cm in length, 4.0 ± 0.45 cm in depth, and 6.8 ± 0.71 cm in diameter [7]. The WS refers to the ability of the spleen to move freely inside the abdominal or pelvic cavity, only being anchored by its vascular pedicle and not to any surrounding organs. This condition can cause complications due to the long pedicle, which can twist on itself. Literature states that the degree of torsion varies from 90° to 2160° [1].

The presentation of a WS is often non-specific, with many asymptomatic patients remaining undiagnosed or discovering their condition incidentally. However, symptomatic patients may exhibit a variety of clinical signs and symptoms, such as vomiting, abdominal distention, a palpable, movable mass in the abdomen or pelvis, or other signs and symptoms of an acute abdomen, as in the case presented. These signs and symptoms may be caused by torsion or detorsion of the splenic pedicle. In severe cases of pedicle torsion resulting in splenic rupture or infarction, the patient can exhibit symptoms of peritonitis and may be in shock due to hemoperitoneum [1].

Medical imaging plays a crucial role in the diagnostic evaluation of WS. Among the array of diagnostic modalities, ultrasonography and abdominal computed tomography (CT) scans are integral tools, as they reveal the absence of the spleen in its conventional anatomical location. Instead, these techniques identify a comma-shaped structure located in alternative abdominal or pelvic regions. [8,9].

Additional imaging methods used for diagnostic purposes include Doppler ultrasound, which assesses blood flow dynamics, magnetic resonance imaging (MRI) providing detailed images without radiation exposure, nuclear scintigraphy to evaluate splenic function, and angiography for visualizing the vascular anatomy of the spleen. Diagnostic laparoscopy further facilitates direct visualization of the spleen's location and condition. [8,10].

The etiology of WS remains multifaceted and not fully understood. It is believed to result from conditions that weaken splenic ligaments, including congenital anomalies such as absent or underdeveloped ligamentous supports, and acquired laxity due to hormonal changes, multiparity, or trauma [11,12]. Recent studies underscore a complex interaction of genetic predispositions and anatomical variations in splenic vasculature and ligamentous attachments as contributing factors [13,14]. For instance, genetic studies have shown that certain mutations increase susceptibility to WS by up to 30 % in affected populations [13]. Additionally, anatomical variations in splenic vasculature, such as anomalous arterial routes, have been identified in over 50 % of WS cases [14].

Our case presented without identifiable predisposing conditions, highlighting its unique nature within WS manifestations. Advances in imaging have significantly improved early detection and understanding of WS, revealing diverse clinical presentations and underlying anatomical abnormalities.

Patients who are asymptomatic with a WS should undergo surgery due to potential complications. Conservative management is discouraged because of high complication rates, which exceed 50 % [1,15].

The surgical approach for WS depends on the condition of the spleen and the patient's symptoms. Splenopexy involves repositioning and securing the spleen in the abdomen, which is ideal for viable spleens to prevent torsion and reduce the risk of overwhelming post-splenectomy sepsis [16]. This procedure can often be performed laparoscopically, ensuring minimal invasiveness and quicker recovery, thereby minimizing hospital stay. However, for significantly enlarged spleens, open surgery may be preferred [17].

In cases where the spleen is non-viable or severely torsed, splenectomy is advantageous. Although it can be done laparoscopically, open surgery is preferred for unstable patients or when dealing with a markedly enlarged spleen [1,15].

During emergencies such as peritonitis, urgent laparotomy is necessary to assess and address intra-abdominal issues promptly. Subsequent consideration of laparoscopy depends on hospital capabilities and patient stability, aiming to minimize trauma and expedite recovery [16,17].

## 5 Conclusion

WS presents a diagnostic challenge requiring high clinical suspicion, particularly in cases of chronic, intermittent abdominal symptoms. This case underscores the significance of timely diagnosis in WS, where symptoms persisted undiagnosed for over a decade due to its rarity and non-specific presentation. The prolonged course of undiagnosed WS in a 45-year-old woman highlights the necessity for thorough diagnostic evaluations and a heightened index of suspicion among clinicians. The case highlights the critical role of imaging in confirming diagnosis and preventing complications. Surgical intervention remains the definitive treatment, given the risks associated with conservative management. This unique presentation contributes valuable insights to medical literature, stressing the importance of considering WS in the differential diagnosis of abdominal pain.

During the preparation of this work the authors used ChatGPT in order to improve language and readability with caution. After using this tool, the authors reviewed and edited the content as needed and take full responsibility for the content of the publication.

## Informed consent

Informed writing consent for publication, including the use of images, was obtained from the patient.

## Ethical approval

This case report did not require ethics approval as it involves a single patient case that does not constitute research according to our institution's guidelines. The Author Form Institutional Review Board (IRB) of Sudan Medical specialization board has confirmed that ethical approval is not necessary for case reports of this nature. Informed consent for publication, including the use of images, was obtained from the patient.

## Funding

No extramural funds were used to support this case report.

## Author contribution

All authors have read and approved the published version of the manuscript.

## Guarantor

Alsadig Suliman accepts full responsibility for the work and the conduct of the study, had access to the data, and controlled the decision to publish.

## Research registration number

Not applicable – this submission is a case report and not a registered study.

## Conflict of interest statement

The authors declare no conflicts of interest. The authors confirm the accuracy of the data.
